# Editorial

**Published:** 1856-10

**Authors:** 


### Editorial.

### SALUTATORY.

In assuming responsibilities in a new and untried field of labor, there is naturally some misgiving—some doubt about the result—*something* that will not permit the mind to be wholly at peace; and yet there is often, at the same time, an under-current impelling the mind to believe that it can, and determine that it will, overcome all obstacles to success. With such doubts and such confidence, we have taken charge of the Register. Our doubt arises from a knowledge of our incompetence and inexperience—our confidence, from rectitude of purpose, and an assurance of the hearty good-will and co-operation of our professional brethren, who have so nobly sustained the Register in its past career.

In the last number, we issued a "Prospectus," setting forth, to some extent, our hopes and intentions, and we trust the present issue is *the beginning of their fulfillment.* For any mistakes and oversights, we ask the indulgence of the reader. The business is new to us, and we expect to improve. We intend that, for neatness and accuracy, the "Register" shall be equal to any professional journal extant; and, if we become convinced that we can not accomplish this, we will endeavor to place it in the hands of those who can.

To those who have so promptly and kindly contributed to our pages, we return our most hearty thanks. It will ever be our ambition to keep up an original department that will be an honor to the profession, an earnest of its future triumphs, an ornament to its literature, and an evidence of the progressive energy and scientific attainments of our patrons. To accomplish this, we are aware that we must rely exclusively on the "free-will offerings" of our brethren, as our patronage will not justify a pecuniary outlay for matter to fill our pages; yet we feel assured that this confidence is not misplaced, as, in every good cause, volunteers are more reliable than hired forces.

While we intend to express ourselves freely and fearlessly in all cases, we ask no more for our opinions than they are intrinsically worth. We hope each reader is a freeman, and thinks for himself. And while it will ever be our aim to be *truthful*, we will make no special effort to be *consistent;* but if, in the increasing light of professional science, the opinions expressed yesterday are found to be erroneous to-day, they will be condemned as freely as though we had never uttered them.

To our editorial brethren it does not become us to say any thing. We send them this number as our introductory card. Should they permit the desired acquaintance, we hope to be able to conduct ourselves toward them with all due courtesy, and with that propriety which should ever characterize the fraternity.

But it is folly for us to spend time in telling you what we hope, or intend to do. Subscribe for the "Register," if your judgment will allow, and then you can not only judge of our success, but aid us in pushing forward that great work in which we are alike interested. TAFT & WATT.

---

### TO THE FRIENDS OF THE REGISTER.

We wish, as intimated in our "Prospectus," to enlarge the Register as soon as practicable. In order to this, it is necessary to have an increased circulation, and a larger amount of original communications. Those who desire the West to have a journal worthy of itself—a journal equal to the wants of the great profession in the West—will at once know what is to be done. Can you not, then, each obtain *one* new subscriber? Do this, and you will at once obtain a Dental Journal equal in size to any extant, and at the present price. We hope that all our friends will endeavor to extend our circulation, and that all who have any thing to communicate will write for our pages without reserve. Many not in the habit of writing—indeed, many who have never written for the public—need only begin, and they will be surprised at their success. To such we would say, Send us every professional item in which you feel an interest, and you will find that others will be interested with you.

We intend, as soon as practicable, to issue the "Register" a month earlier than at present dates. Three of the quarterlies are now issued simultaneously. Those who read all of them would, no doubt, rather receive them in regular rotation than all at once. We will endeavor to have our next number out by the first of December.

---

### TO THE PROFESSION.—PRACTICAL DENTISTRY.

Interchange of opinion and practice is a valuable means of improvement, and one that has been sadly neglected. The older and better class of operators bestow much time and labor upon their operations, and usually have much to do, and thus they excuse themselves. Those younger in years and practice, though they may not be so pressed with business, think that it is the duty of those of more professional experience, to write and speak of practice in its details; and thus they excuse themselves. Those who have kept themselves shut up in some sequestered spot, isolated from their professional brethren, think,—"I never got much from the profession,

—I worked mine out *myself;* and I am not going to incommode myself to give anything"; and so they excuse themselves.

Now, be kind enough to lay aside all these excuses, and give us your methods of practice. Let each one give, clearly and concisely, the manner of performing his common every-day operations. Do not aim to cover a great amount of ground. Take, for instance, a single filling, and describe every step of the operation. We hope every one who considers himself an operator, will begin a course of this kind immediately. Make your articles short and concise. Do not stop to inquire if any one else performs it as you do. Describe the instruments you use in each case, and your mode of using them.

Now, gentlemen, let us hear from you. We intend to reserve a nice little corner in the "Register" for these contributions on operative dentistry. We intend, also, to reserve an "Interrogatory Corner," and we solicit questions in regard to difficulties that arise in practice. They will then stand before the profession till some one can render a clear solution of them. T.

---

### TO PHYSICIANS.

Physicians whose patients have not access to a reliable dentist are often at a loss for practical information respecting the diseases of the teeth and mouth. Such information they can obtain more readily and pleasantly from a standard Dental Journal than from any other source. With this feeling, and these views, when engaged in the practice of medicine, we subscribed for "*The Dental Register of the West*," and read it with the same pleasure and profit that we now read a standard medical journal. To a few whom we know to be so situated, and others of well known liberality, we send this number of the "Register," hoping that, if they do not wish to be considered subscribers, they will read it carefully, and then promptly return it by mail, with their names plainly written on it, that we may know whence it is returned. W.

---

### AMERICAN DENTAL CONVENTION.

The convention held a session of three days, which were spent in the discussion of practical subjects, and the investigation of new inventions and improvements. We can only say that the meeting was *intensely* interesting. "The good things of this life" were abundantly dispensed to the members, by Messrs. Jones, White, and McCurdy, in an entertainment at the Astor House, and, by the dentists of New York and vicinity, in a supper at Dodsworth's Assembly Rooms. The entertainments were both according to the hearts of the givers. They need no further, and can receive no higher commendation.

The next meeting will be in Boston. Our western brethren, no doubt, feel disappointed at this; but all the arguments used in favor of the eastern location were highly creditable to the West. The avowed policy seemed to be to go on a mission to those regions which take the least interest in professional progress; and as Boston was not so thoroughly represented as Cincinnati, St. Louis, or Chicago, it was, of course, selected. While we do not believe the profession of Boston have merited such a left-handed compliment, and while we confess ourselves disappointed in regard to the place of meeting, we believe that the sentiment of our western brethren was truly given by Prof. Taylor, who, at the announcement of the vote, arose and stated—"Gentlemen, *we* expect to be *there*." Although we presume, the next meeting will be in the West, we hope it will not be merited (?) by our indifference.

---

### WESTERN DENTAL SOCIETY.

The members of this society, as will be seen by the report of its proceedings, went to work in true western style—"forenoon," "afternoon," and "evening" sessions; just as though the work *must* be done, and none but they could do it. And, western- like, they adjourned to meet in May, a busy month, but one favorable to active thought—thus insuring the attendance of those desiring light rather than ease; not those who are so *busy* they can not afford to go, but those who are so progressive they will not stay away. Glad are we that they do not follow the precedent of *dog-day meetings*. We hope their prosperity will be equal to their energy

---

### PROCEEDINGS OF SOCIETIES.

We have devoted a large portion of our space to this department of the Register. We are glad to have such matter to lay before our readers, and only regret that the best report must fall so far behind the reality. To Dr. Clark, of the "*Obturator*," are we under obligation for an advance proof-sheet of the proceedings of the "Western Society;" and also to the "American Convention," for its liberality in defraying the expenses of reporting its proceedings. The committee furnished us with stereotype plates of the reports, for which we return our sincerest thanks.

---

### AMERICAN SOCIETY OF DENTAL SURGEONS.

Our readers will see that this *pioneer* society is dissolved. We would say, "Peace to its ashes," were it not that its remains have not turned to ashes, but are still either actively employed in the cause of progress, or indelibly stamped on the pages of dental literature. Its men, and their sayings and doings, require no laudation or eulogy.

### OHIO COLLEGE OF DENTAL SURGERY.

The regular course of lectures in this school will commence on Monday, the third of November. We are happy to state that the infirmary is still well patronized, and that engagements are already made for many cases that will require attention in the early part of the session. Students need have no fears in regard to opportunities for practical instruction. The professors are now severally engaged in revising and remodelling their respective courses of lectures, and otherwise preparing for the duties of the campaign. The entire arrangements for infirmary practice will be much amplified and improved. The professor of operative dentistry is rapidly extending his cabinet, both in the way of enlarged models and natural organs. Systematic demonstrations, both in and out of the mouth, will be such as to render the theoretical instructions so clear that it can not fail to be understood. Energetic, ambitious students can do themselves no greater kindness than to aim for the position of "resident dentist" in the infirmary. Of those who desire it, it is given to him who stands the best examination before the faculty and committee.

---

☞ The increase of dental associations is an encouraging evidence of professional harmony and progress. What do you say, brethren, to an association in Cincinnati? Are we ready? If not, suppose we meet and devise a way to get ready. We would be glad to meet *all* our brethren, at least as often as semi-occasionally. Who will take the initiatory steps?

---

### MISSISSIPPI VALLEY DENTAL DEPOT.

The profession in St. Louis and vicinity are exceedingly fortunate in regard to a dental depot. Dr. Leslie's accurate, *experimental* knowledge of metallurgy, his thorough acquaintance with dental science, and his well known industry, designate him as the man for the place. We are glad to learn that he is fully appreciated. See advertisement.

---

### NEW CORUNDUM WHEELS.

We have recently received from Messrs. Jones, White, and McCurdy, a few corundum wheels that, in some respects, excel any thing we have used heretofore. They do not clog and become smooth, as almost all the wheels which we have had, but they retain a clear, sharp, cutting surface all the time, we presume, till they wear out, and that will be a long time, for they are quite as hard, if not harder, than any we have seen before. They cut right along, *requiring no sharpening.* They are made by a new formula, which we think an improvement. The only fault we find is *that they cut too fast.* Be careful or you will spoil your teeth when you begin to use them. *They are self-sharpeners.*

*New Form of Astringent Application.*—By Dr. William Bayes, Brighton.—Pure glycerine dissolves nearly its own weight of tannin, affording a very powerful local astringent application.

The solution of tannin in pure glycerine appears to me to supply a desideratum long felt, and capable of a great variety of useful applications.

The solvent property of glycerine over tannin, allows us to form a lotion of any desirable strength, as the solution is readily miscible with water.

The solution of tannin in glycerine, in one or other of its strengths, is peculiarly applicable to many dissorders of the mucous membrane, readily combining with mucus, and forming a non-evaporizable coating over dry membranes; hence it may with benefit be applied to the mucous membranes of the eye and ear in many of its diseased conditions. It forms a most convenient application to the vaginal, uterine, urethral, or rectal membranes, where a strong and non-irritant astringent lotion is desired.

In local hæmorrhages, where the bleeding surface can easily be reached, it will prove very convenient, and may be applied either with a sponge or small brush.

The solution must be kept in the dark, and should not be prepared for any great length of time before used, or decomposition will occur.

It is singular that glycerine does not possess the same property towards gallic acid.—*Association Med. Jour.*

The above solution of tannin, will be found an admirable application for inflamed lips, giving prompt relief in many cases, and in nearly all, facilitating the cure. Its strength may be varied according to the circumstances of the case. Its use will not be followed by that distressing dryness of the membranes, that often results from other astringent preparations. W.

☞ It is confidently expected that, if any who receive this number of the *Register* do not wish to be considered subscribers, they will promptly return it, carefully marked, that we may know by whom it is returned.

As we intend, if possible, to issue the next number of the "Register" by the first of December, advertisers and contributors will please send in their articles immediately. All articles for publication should be received by us at least one month previous to the date of the number in which they are expected to appear.

